# Partial Sacrectomy With Lumbopelvic Fixation for Sacral Giant Cell Tumor: Emphasizing Optimal Neuro-Oncological Outcomes

**DOI:** 10.7759/cureus.71602

**Published:** 2024-10-16

**Authors:** Sarang Gotecha, Ashish Chugh, Prashant Punia, Ramis Aziz, Jayant Gaud, Ishant Rege

**Affiliations:** 1 Neurosurgery, Dr. D. Y. Patil Medical College, Hospital & Research Centre, Dr. D. Y. Patil Vidyapeeth (Deemed to be University), Pune, IND

**Keywords:** embolization, giant cell tumor, pedicle screws, sacrum, lumbosacral region

## Abstract

Giant cell tumors (GCTs) of bone are classified as intermediate malignant tumors with a significant potential for local infiltration. Despite their benign histopathological appearance, these tumors exhibit extreme local aggression. The sacrum is the most commonly affected spinal region, followed by the lumbar, cervical, and thoracic regions. Active treatment of sacral GCTs is essential for preserving the sacral nerve roots and filum, allowing patients to maintain an impairment-free life. While excision of sacral GCTs enhances local control, it poses challenges, including the risk of losing bowel and bladder control. In this report, the authors share their experience with lumbopelvic fixation using gluteus maximus flaps to address the dead space following partial sacrectomy for sacral GCT in a 35-year-old female, ultimately achieving an optimal neuro-oncological outcome.

## Introduction

Giant cell tumors (GCTs) are rare benign bone tumors, accounting for less than 10% of all benign bone tumors. They are characterized by fusiform to ovoid mononuclear stromal cells and osteoclast-like giant cells, commonly affecting the epiphyseal regions of long bones [1]. GCTs are most frequently diagnosed in individuals aged 20-40 years, with a predominance in females [2]. An estimated 2-15% of all GCTs are spinal GCTs, with approximately half of these being sacral GCTs [3]. The majority of GCTs are clinically silent and discovered incidentally.

Sacral GCTs, although benign, are particularly aggressive and are often diagnosed late, as initial symptoms can be mistaken for sciatica [4]. Resection of sacral tumors is challenging due to their often considerable size and proximity to critical anatomical structures [5]. The tumor typically encases sacral nerve roots and can be extensive within the bone [4]. Sacral tumor resection has consistently been associated with high failure rates, with local recurrence rates reaching up to 75% [6]. While excising the sacral GCT can improve local control, removing the involved sacral segment may lead to loss of bowel and bladder control. Additionally, managing the instability resulting from the removal of vital spinal structures presents further challenges.

Radiotherapy and arterial angioembolization have been utilized as alternative therapies for sacral GCTs in cases where excision may cause significant functional impairments [7-9]. In this article, we discuss our experience with lumbopelvic fixation using gluteus maximus flaps to address the dead space following partial sacrectomy.

## Case presentation

A 35-year-old female presented to the department of neurosurgery with complaints of low backache radiating to both lower limbs for two years, which had aggravated over the past three months. She also experienced associated tingling and numbness. Examination revealed sacral hypoesthesia and mild weakness in bilateral knee flexion. A rectal examination showed a large, globular, firm bulge at the posterior rectal wall.

**Figure 1:**
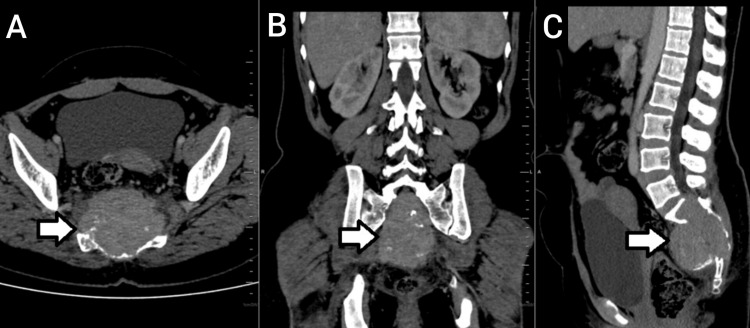
Lumbopelvic CT scan demonstrating a large, well-defined expansile lytic lesion (arrow) involving the sacrum (S1-S5) with extension into the presacral and sacral spaces, presented in (A) axial, (B) coronal, and (C) sagittal views

**Figure 2:**
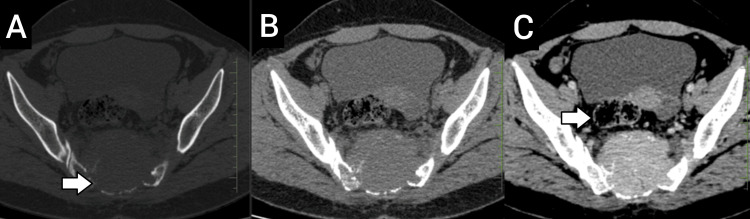
Lumbopelvic CT scan in axial view showing the lesion with destruction of the pelvic surface of the sacrum: (A) bone window, (B) non-contrast scan, and (C) contrast-enhanced scan, demonstrating slight anterior displacement of the rectum

The CT scan revealed a large, well-defined, expansile lytic lesion involving the sacrum (S1-S5) with extension into the presacral and sacral spaces, exhibiting contrast enhancement. The lesion measured approximately 5.8 × 6 × 7 cm (CC × AP × TR) (Figure 1). Several hyperdense foci within the lesion were identified as destroyed bone fragments. The lesion extended anteriorly, causing destruction of the pelvic surface of the sacrum and slight anterior displacement of the rectum while maintaining the fat planes. It involved the sacral canal, sacral crest, and the dorsal surface of the sacrum posteriorly. Laterally, the lesion extended up to the articular surfaces of the sacroiliac joints bilaterally, eroding the articular surface on the right side. Extensive soft tissue infiltrates, showing post-contrast enhancement, were noted bilaterally along the piriformis muscles, accompanied by loss of the intervening fat planes (Figure 2). Additionally, extensive surrounding fat stranding was observed. A linear fracture was noted in the medial part of the sacrum on the right side, involving the S5 vertebra.

**Figure 3:**
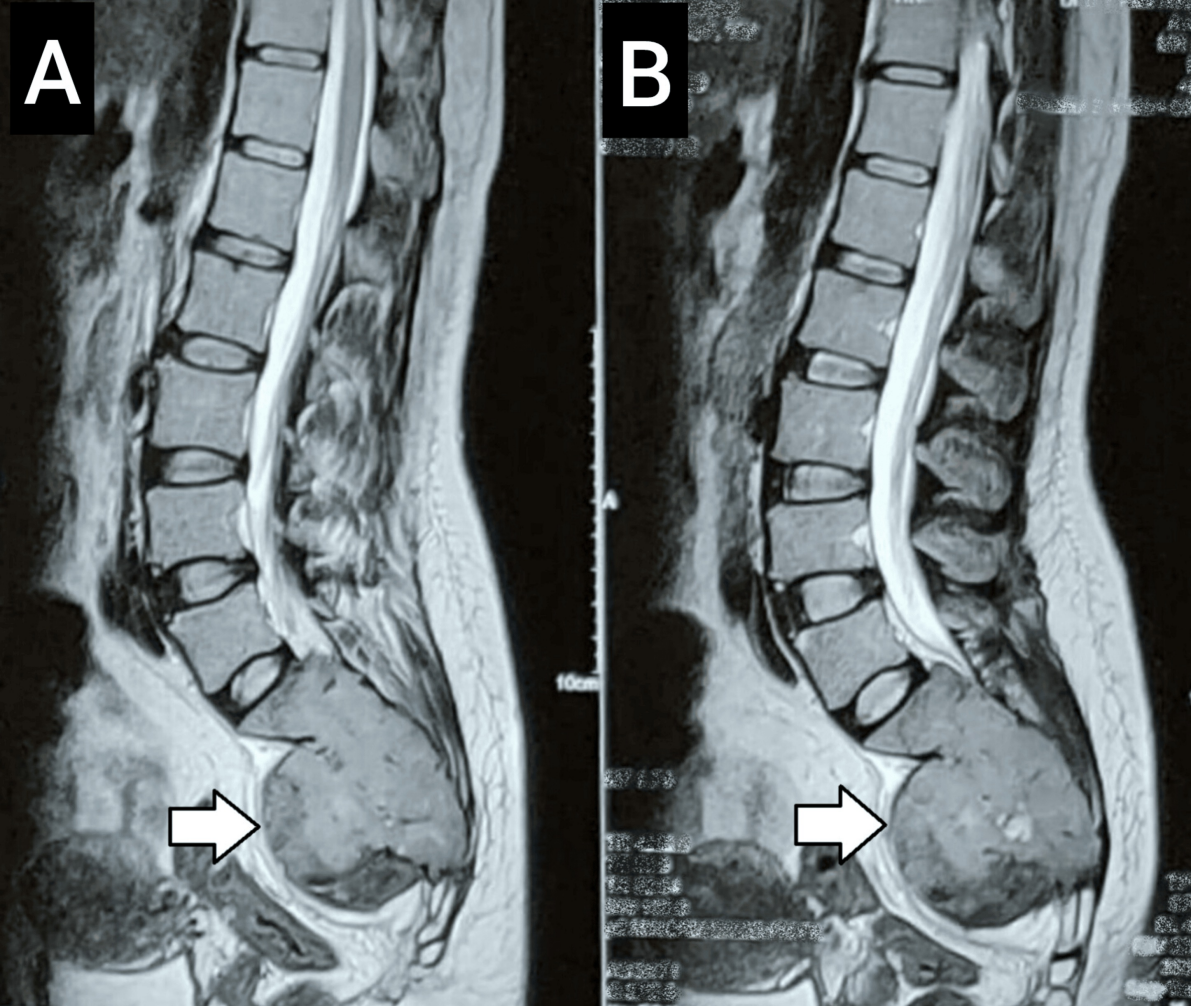
Lumbopelvic MRI in sagittal view demonstrating a lobulated mass lesion (arrow) centered at the sacrum, causing destruction of the sacral bone: (A) non-contrast scan and (B) contrast-enhanced scan

Her MRI revealed a lobulated mass lesion centered in the sacrum, causing destruction of the sacrum (Figure 3). On T1-weighted images, the lesion appeared hyperintense compared to the muscles. It was hyperintense on short tau inversion recovery images and iso- to hyperintense on T2-weighted images. In the post-contrast study, the lesion exhibited heterogeneous enhancement with non-enhancing necrotic areas.

The patient was positioned prone, and a posterior linear midline incision was made from L4 to S5. Once the tumor was visualized, a biopsy was performed, and decompression was initiated. However, the tumor was highly vascular, leading to the abandonment of the procedure due to massive intra-tumoral bleeding and hemodynamic instability. Hemostasis was achieved using packing mops and hemostats, and she was transfused with four units of blood simultaneously.

**Figure 4:**
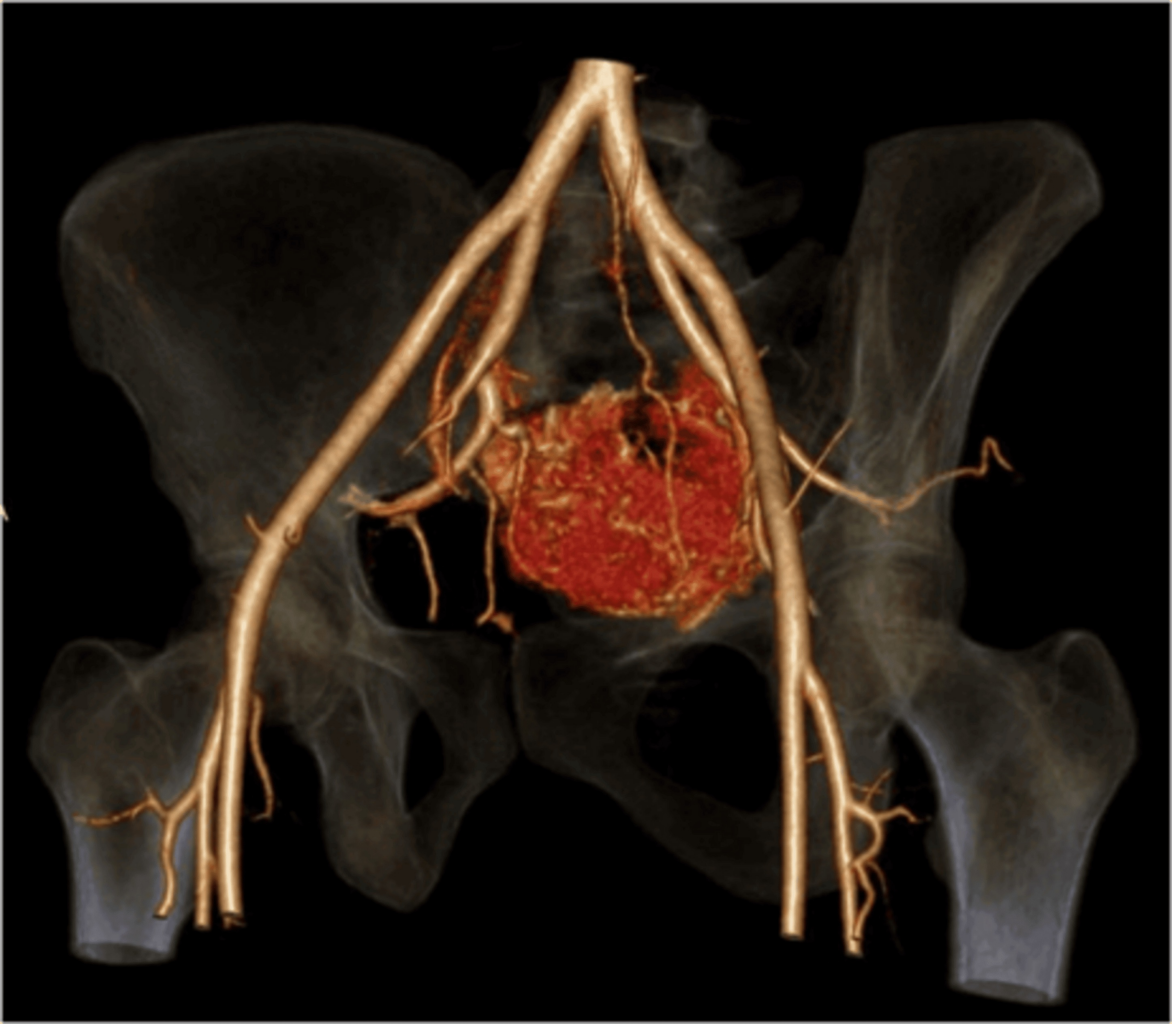
Preoperative planning using a 3D reconstruction of the CT angiogram Anterior view of the lumbopelvic region illustrating that the mass appears to receive arterial supply from both the bilateral sacral arteries and the median sacral artery, along with adjacent vital structures such as the internal and external iliac arteries.

Postoperatively, there were no deficits, and she was stabilized hemodynamically in the intensive care unit. The histopathology report of the biopsy was suggestive of a GCT. An interdisciplinary meeting was held with teams from neurosurgery, surgical oncology, plastic surgery, and interventional radiology. The CT angiogram revealed that the mass derived its arterial supply from the bilateral sacral arteries and the median sacral artery (Figure 4). Interventional radiology-guided embolization of the median sacral artery and bilateral lateral sacral artery was subsequently performed.

**Figure 5:**
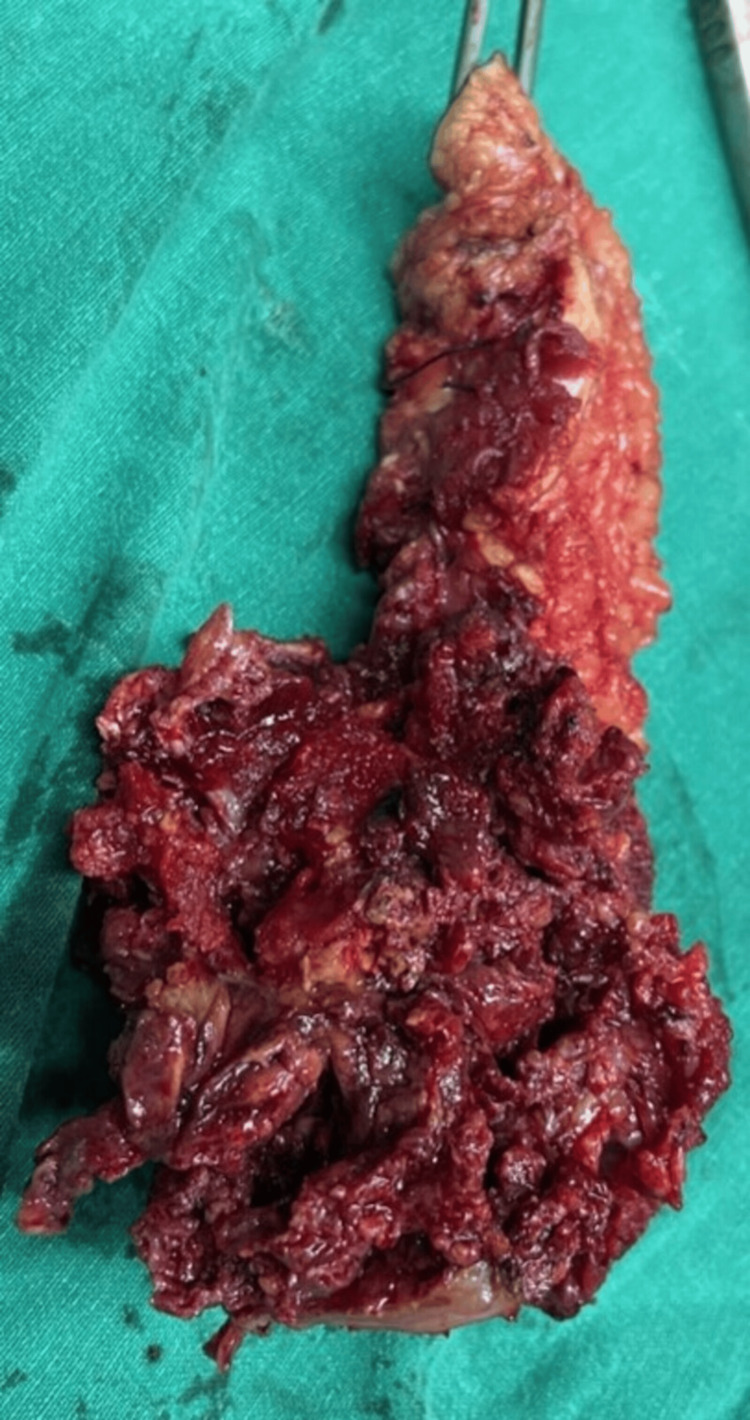
Excised tumor specimen

**Figure 6:**
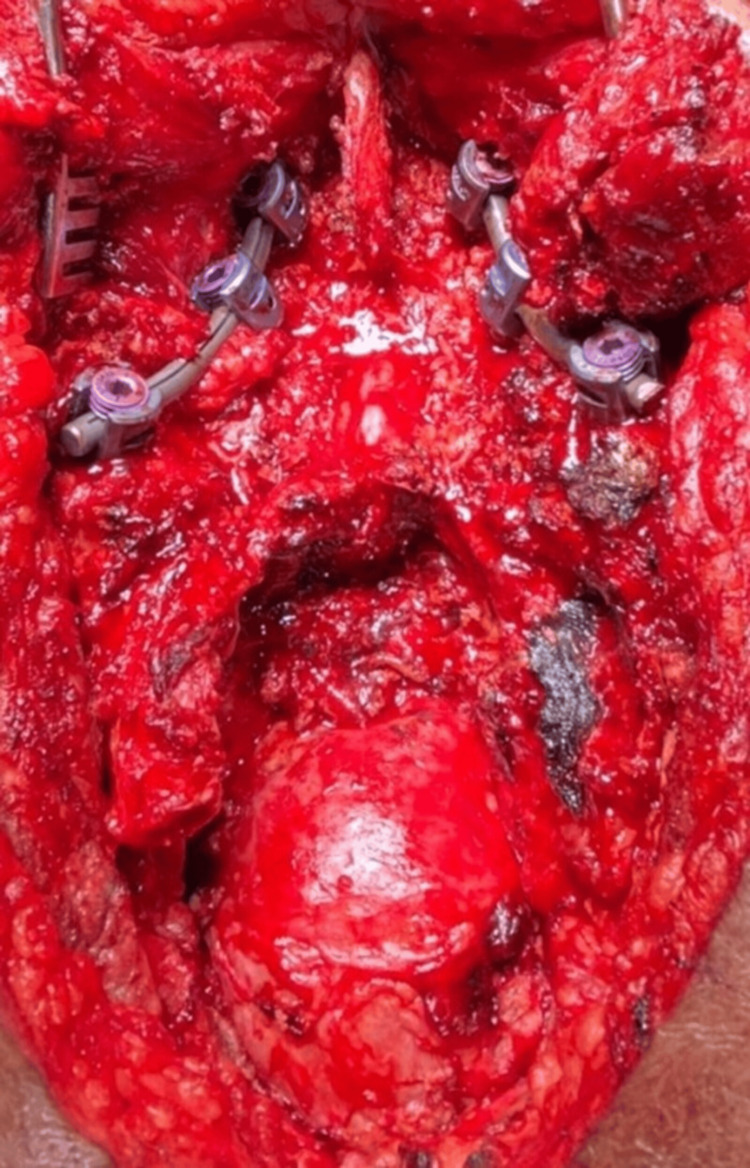
Intraoperative image displaying the excised tumor cavity, decompressed dura, and the fixation with the placement of implants

**Figure 7:**
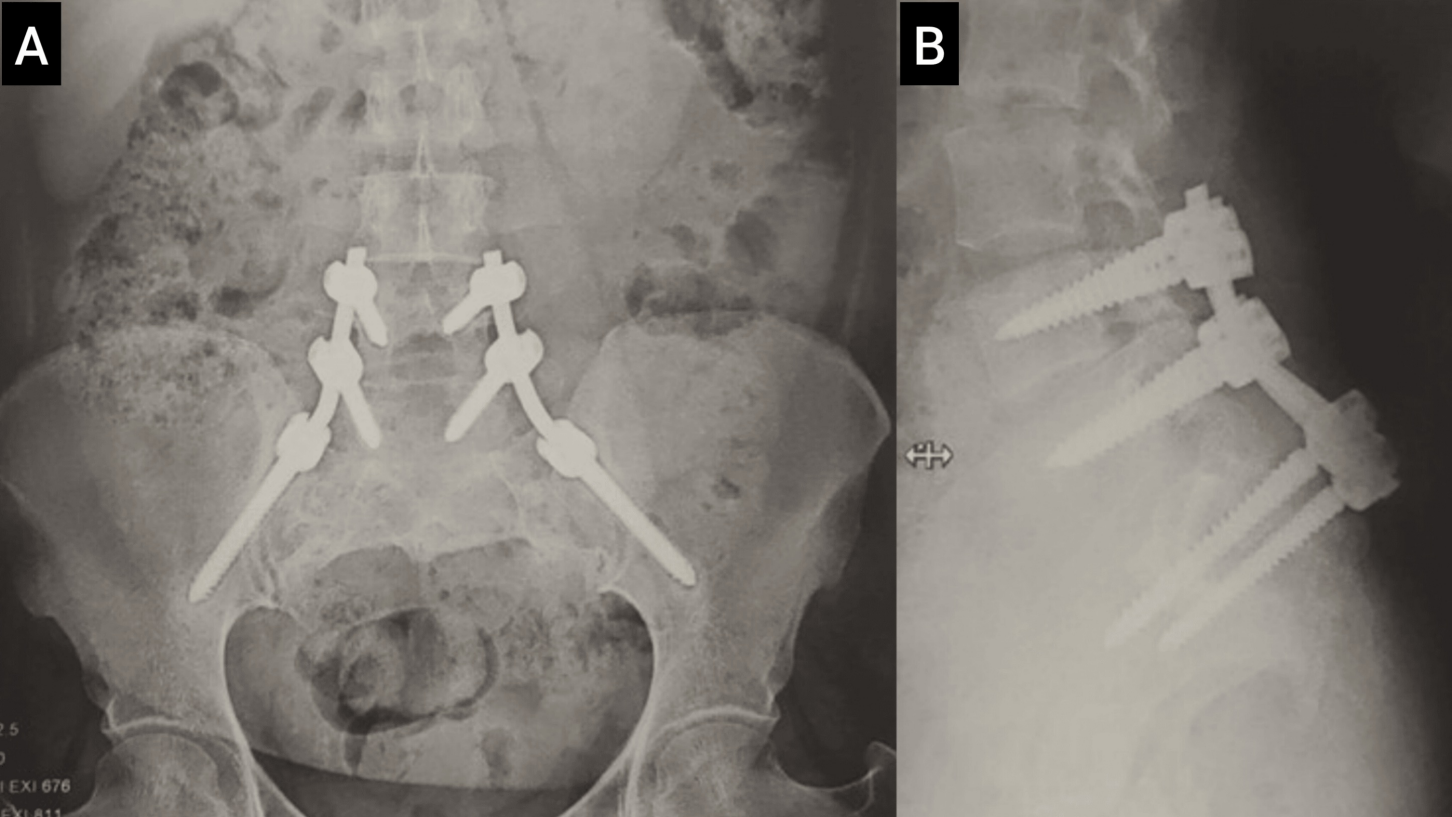
X-ray of the lumbosacral spine: (A) anteroposterior view and (B) lateral view, illustrating the fixation and placement of implants

On the eighth postoperative day, she was taken for reexploration with the assistance of oncosurgery and plastic surgery. A partial sacrectomy was performed up to the mid-S3 level, along with curettage of the lesion from the S1 and S2 levels (Figure 5), after which lumbopelvic fixation was completed (Figure 6, Figure 7). Closure was achieved using a bilateral gluteus maximus musculocutaneous flap. Postoperatively, she developed weakness in knee flexion, and her sacral hypoesthesia worsened.

Her urinary catheter was retained and removed after one and a half months, during which she subsequently passed urine. She was advised to start denosumab (a monoclonal antibody used to treat GCTs of the bone, bone metastases, treatment-induced bone loss, and osteoporosis), but she refused the treatment. Her scans were repeated at intervals of six months, one year, and two years, and they did not show any signs of recurrence. During her routine checkup two months ago, she was pain-free and ambulatory without assistance.

## Discussion

GCTs of bone, constituting approximately 5% of all primary bone tumors, are classified as intermediate malignant lesions with a significant capacity for local infiltration. Data indicate that the sacrum accounts for 6-9% of all GCTs, with a predominant diagnosis occurring around the age of 30 years [10]. Although these tumors exhibit benign histopathology, they are locally aggressive [9].

Sacrococcygeal tumors are often diagnosed only after substantial growth, as the sacral canal and pelvis can adapt to enlarging lesions [11]. Initial diagnoses are frequently delayed due to the latent onset of symptoms associated with tumors in this region, including urinary incontinence and radicular pain.

A comprehensive investigation utilizing contemporary imaging techniques is essential, as the tumor may alter anatomical landmarks and compromise surrounding bone structures. Given the severity of the disease and the complex anatomy of the pelvis, a multidisciplinary approach involving a neurosurgeon, onco-surgeon, oncologist, plastic surgeon, physical therapist, and psychiatrist is often necessary. Preoperative planning in this multidisciplinary setting facilitates the development of various surgical approaches and the optimal integration of techniques. Each surgical specialty provides unique insights into sacrococcygeal anatomy that can enhance patient care. Treatment options include wide local excision or radical resection following embolization, as opposed to partial excision while preserving essential bodily functions [12].

To mitigate intraoperative bleeding, bilateral iliac artery ligation or arterial embolization is recommended. Internal iliac artery embolization reportedly achieves a 78% success rate [13]. For sacral GCTs located below the S3 level, radical excision is considered the most appropriate surgical intervention. Research indicates that normal bowel function is maintained in all patients undergoing bilateral S4-S5 resection with at least one S3 nerve root intact, while normal bladder function is preserved in 69% of these cases [14]. Preserving the bilateral S3 nerve roots is crucial for normal bowel and bladder function. Several studies have demonstrated that sacral GCTs can be effectively treated with nerve-sparing surgery, resulting in acceptable local control. This approach involves tumor curettage in the cephalad region (above the S3 level) and en bloc excision in the caudal region (at or below the S3 level) [12]. Patients who underwent nerve-sparing surgery alone exhibited a 29% local recurrence rate [15].

In cases involving large sacral GCTs, intraoperative neuromonitoring (IONM) is often technically challenging due to the complex anatomy of the sacral region, which includes extensive bone involvement and potential neural compression. This complexity may hinder the ability to obtain reliable signals for monitoring motor and sensory pathways, particularly in instances of significant tumor invasion into the sacral bone. IONM techniques such as motor-evoked potentials and somatosensory evoked potentials can be adversely affected by the tumor’s size and location, which may distort normal nerve anatomy and function. Distorted signals or the inability to maintain stable neuromonitoring can result in unreliable outcomes, making IONM less effective for ensuring neural preservation during surgery.

The objective of treating sacral GCTs is locoregional control, and the use of IONM may impede the extent of resection of the lesion. Given these challenges, surgeons may opt not to utilize IONM for certain large sacral tumors, particularly when technical limitations outweigh potential benefits.

There is a considerable risk of morbidity following total and partial sacrectomy, which can result in motor, urinary, bowel, and sexual dysfunctions. Lumbopelvic instability can also arise depending on the extent of sacral removal. After sacral tumor excision, preserving the sacroiliac joint is crucial for maintaining stability between the spine and pelvis. Therefore, determining surgical margins, the extent of resection, and the potential sacrifice of nerve roots are essential in predicting the patient’s functional outcome [16]. The frequency and severity of bladder and bowel dysfunction increase with cranial sacrectomy levels, prolonging hospital stays. The extent of surgical resection should be greater for locally aggressive tumors to achieve long-term disease control. The potential benefits and risks associated with block resection should be thoroughly discussed with the patient, as intralesional surgery or adjuvant treatments may not achieve adequate local disease control. Extensive tumor resection allows for a stable spine, preservation or restoration of neural function, and prevention of tumor recurrence, along with decompression of the cord and nerve roots during spinal fusion procedures. Fixation post-tumor removal helps restore or maintain normal gait, alleviate neurological impairments, and reduce pain [6].

The pedicle screw-rod construct is the latest generation of instrumentation used in spinopelvic reconstruction. Compared to earlier constructs, this system is easier to position, safer, and provides sturdier fixation. The polyaxial screws’ lumbopelvic fixation offers a more rigid attachment and is safer for instrumentation, along with iliolumbar fixation that is biomechanically stable. Initially, monoaxial or polyaxial pedicle screws are placed in the lumbar spine, while polyaxial screws are inserted in the ilium.

Typically, the posterior superior iliac spine (PSIS) serves as the site for iliac screw insertion. The trajectory of the iliac screws is directed toward the anterior inferior iliac spine. A notch is created on the dorsal cortex of the PSIS using a rongeur. An awl is then employed to create a tract along this trajectory, extending to the far cortex. Intraoperative C-arm imaging is utilized to confirm the trajectory of the tract. Good purchase and stability are achieved with iliac screws measuring up to 141 mm in length for men and 129 mm for women [17,18]. The screws most commonly used have a diameter of 7.5 mm [19,20]. Following this, vertical rods are segmentally affixed to the lumbar spine after bending, and then they are connected to the ilium. Transverse rods may also be added to enhance stability around the horizontal axis. The primary advantage of using polyaxial pedicle screws is their capacity to provide three-dimensional stability [6].

Although radical excision reduces the likelihood of local recurrences, it necessitates the sacrifice of the sacral nerve root and may require lifelong colostomy and catheterization. Recent treatments for this condition have also included bisphosphonates, denosumab, and interferon [12].

## Conclusions

Sacral tumors require thorough preparation and advanced imaging techniques, such as a CT angiogram. Sacral GCTs should be actively treated with the aim of preserving the sacral nerve roots and filum to enable patients to lead a life free from impairment. It is essential to provide appropriate counseling to patients, ensuring they have realistic expectations regarding functional outcomes. For lesions located at or above the S3 level, nerve-sparing surgery should be performed through a partial sacrectomy starting from the mid-S3 level, accompanied by curettage above. In contrast, en bloc resection is recommended for lesions situated below S3.
